# The interactive effect of ambient temperature and brood size manipulation on nestling body mass in blue tits: an exploratory analysis of a long-term study

**DOI:** 10.1186/s12983-022-00456-x

**Published:** 2022-02-28

**Authors:** Aneta Arct, Szymon M. Drobniak, Anna Dubiec, Rafał Martyka, Joanna Sudyka, Lars Gustafsson, Mariusz Cichoń

**Affiliations:** 1 Institute of Systematics and Evolution of Animals, Polish Academy of Sciences, ul. Sławkowska 17, 31-016 Kraków, Poland; 2 Department of Animal Ecology/Ecology and Genetics, Evolutionary Biology Centre, Uppsala University, 752 36 Norbyvägen 18 DUppsala, Sweden; 3 Institute of Environmental Sciences, Jagiellonian University, Gronostajowa 7, 30-387 Kraków, Poland; 4 Evolution and Ecology Research Centre, School of Biological, Environmental and Earth Sciences, University of New South Wales, Kensington, 2052 Australia; 5 Museum and Institute of Zoology, Polish Academy of Sciences, Wilcza 64, 00-679 Warszawa, Poland; 6 Institute of Nature Conservation, Polish Academy of Sciences, Mickiewicza 33, 31-120 Kraków, Poland

**Keywords:** Reproductive effort, Climate change, Birds, Weather conditions, Post-hatching rearing conditions

## Abstract

**Background:**

Relatively few studies have examined the interactive effects of ecological factors on physiological responses in wild animals. Nearly all of them have been short-term investigations that did not include experimental manipulations, limiting our ability to understand how climate change will affect natural populations. Using a 10-year brood size manipulation experiment in wild blue tits (*Cyanistes caeruleus*), we quantified the impact of weather conditions and brood competition on the body mass and structural size (tarsus length) of nestlings just prior to leaving the nest.

**Results:**

We found that variation in nestling body mass on day 14 after hatching was explained by an interactive effect between average ambient temperature experienced during nestling period and brood size treatment. Specifically, in control broods nestling body mass was correlated with temperature in a non-linear manner (concave) with the vertex point (maximum body mass) at ca. 13 °C. In contrast, in enlarged broods nestling body mass permanently increased (also non-linearly) as temperature advanced.

**Conclusions:**

Our results highlight the importance of considering the effects of brood rearing conditions alongside other environmental factors experienced during growth while investigating early-life environmental effects on body condition.

**Supplementary Information:**

The online version contains supplementary material available at 10.1186/s12983-022-00456-x.

## Background

Abiotic factors are key elements of environment which may significantly affect early development and can have profound effects on animal populations and the evolution of life-history traits [1, 2]. Abiotic factors may be particularly influential in case of oviparous animals, such as birds, because these factors can directly act on their development. In consequence, many examples of how weather conditions experienced during early development affect offspring phenotypes originate from studies on free-living birds [3–6]. Passerine birds provide ideal model organisms for addressing this issue, because their reproductive biology has been extensively studied [7–9]. In many species, particularly in nest-box breeding populations, all or part of a bird’s nesting attempt, including the building, egg laying, incubation, nestling growth, and fledgling stages can be easily monitored [10].

Birds may experience a large range of environmental conditions during breeding with respect to local weather conditions. In case of altricial birds, one of the most energetically expensive periods in their life cycle is a time when young are in the nest. While the nestling period is commonly costly for parents as they need to provision for the offspring [11], the young birds face energy limitations because of their rapid growth and development [12]. In particular, adverse weather conditions are expected to result in impaired nestling development due to several reasons. First, the development can be constrained by temperature-dependent physiology and metabolism of the offspring. Outside the optimal range of ambient temperatures (in cold and very hot weather), costs of thermoregulation are higher, thus resources normally allocated to growth have to be traded-off with body temperature maintenance [13, 14]. Extreme weather conditions can be particularly relevant during later nesting stages: females reduce their presence in the nest and in cold weather nestlings have to upregulate their body temperature on their own, whereas in hot weather, the temperature in the nest rises due to the presence of siblings (especially in large broods and in nest-boxes which provide poor insulation from external temperatures) [15]. Second, ambient temperature can modulate parental effects. In the event of low resource abundance and/or weather perturbations, there is an inherent trade-off between self-maintenance vs. parental investment in the current brood [16]. Low temperatures likely increase the costs of self-maintenance in adults because their thermoregulatory costs increase. Moreover, prior to developing own thermoregulatory system, nestlings are dependent on parental brooding to maintain appropriate body temperature, and therefore low temperatures require parents to spend more time brooding [17]. All these investments reduce time available for foraging and can cause decrease of parental investment into the brood in low and very high temperatures. Third, ambient temperature is a direct and important driver of insect abundance (food availability) which decreases during cold seasons [18]. Impaired food quality and quantity can have profound effects on nestling growth [18]. Finally, temperature is an important determinant of ectoparasite prevalence for both parents and nestlings [19, 20]. In cold conditions higher prevalence of fleas is expected and in hot blowflies which both can cause growth retardations [19].

Indeed, growth rate, body size, fledgling success and survival of nestlings have been shown to be influenced by local weather conditions (i.e. ambient temperature) during post-hatching stage [21–23]. However, recent studies suggest that the overall effects of ambient temperature on offspring fitness-related traits are complex (i.e. both positive and negative relationships can occur, depending on species and/or population) and context-dependent (i.e. differing between environments) [21, 24–27]. One explanation of this variability may result from the fact that negative effects of adverse weather can be moderated by adjustments in parental care strategies [24, 28, 29]. Parental care is known to be flexible, changing in response to environmental conditions and the needs of the offspring [30]. Parents are able to assess conditions experienced by their offspring and adjust their parental care accordingly, i.e. parents can mitigate the food shortages by increasing their foraging effort or substituting the most nutritious food items [31]. Plastic shifts in parental care may therefore play an important role in mitigating the negative effects of adverse weather conditions on developing offspring.

Brood size manipulation treatment is a simple and common experiment, in which the number of offspring is experimentally altered in randomly chosen broods, which results in varying levels of parental effort and environmental conditions experienced by offspring (sibling competition) as compared to control broods with the original brood size retained (reviewed in [32, 33]). The expectations are clear: in the absence of parental responses to the experiment, offspring from increased or reduced broods are expected to experience lower or higher parental investment respectively per individual and increased or decreased competition respectively with their siblings, resulting in lower or higher performance respectively (such as body mass, survival, etc.) in comparison to the control broods. While the effects of reducing original broods are not always straightforward [34], several studies showed that parents raising more young than their original brood were faced with a diminished fitness return from each of these young, as lower weight and higher mortality at fledging were observed in experimentally enlarged broods [33]. As such, brood size manipulation is a well-tailored way to experimentally test if environmental circumstances in the nest interact with the influence of weather conditions experienced during growth on body mass and size of nestlings at fledgling.

Here, we present an exploratory research analysis on a long-term dataset from a nest-box population of blue tits (*Cyanistes caeruleus*), for which the brood size manipulation experiment was carried out for at least 10 years. Specifically, we tested the combined effects of weather conditions during nestling stage and brood size treatment on offspring body mass and tarsus length—a measure of structural size—just prior to leaving the nest. We expected nestlings originating from control nests to perform better in comparison to nestling originating from experimentally enlarged broods under relatively unfavourable weather conditions (low temperature and high daily sum of precipitation). More formally, we expected a significant interaction between weather conditions index (average daily temperature and daily sum of precipitation) and experimental treatment.

## Results

Nestling body mass on day two after hatching did not differ either between offspring assigned to control and enlarged broods (F_1, 477.5_ = 0.05, P = 0.83, N = 2905 nestlings) nor between female and male offspring (F_1, 2689.1_ = 1.18, P = 0.28, N = 2905). Hatching date and original brood size also did not affect the body mass of two-day-old nestlings (all P > 0.09).

**Fig. 1:**
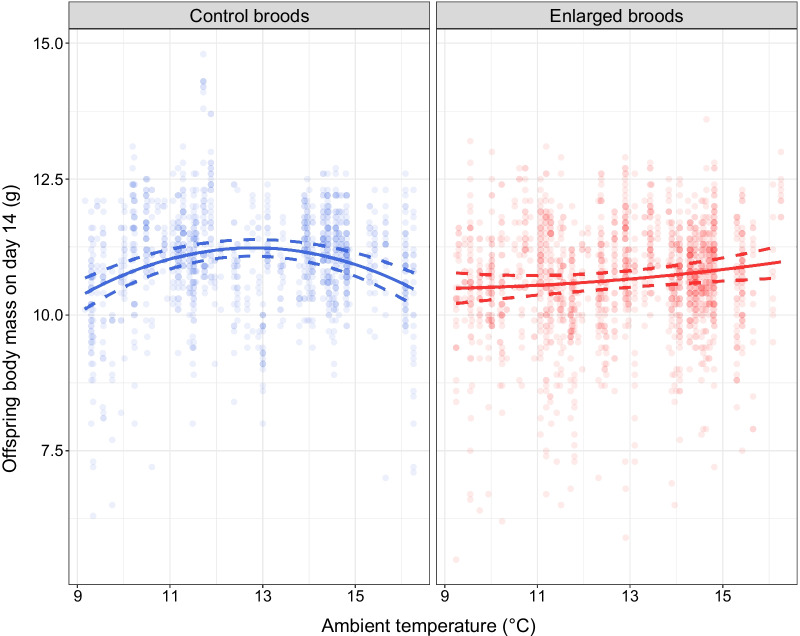
The interactive effect of brood size manipulation and ambient temperature (quadratic term) on nestling body mass 14 days after hatching. The fitted regression lines accompanied with 95% CIs are presented, based on predicted values from the final model (Table 1, blue—control broods, red—enlarged broods)

We found that variation in body mass of 14-day-old nestlings was significantly explained by nestling sex, hatching date, nestling body mass on day two, nestling tarsus length on day 14th and an interaction between brood size manipulation and ambient temperature (Table 1, see full model Additional file 1: Table S2). On day 14 after hatching body mass of female offspring was lower than male offspring (mean ± SE; 10.57 ± 0.02 g vs. 11.02 ± 0.03 g, respectively; Table 1).The interactive effect resulted from the fact that the relationship between offspring body mass and temperature showed a concave pattern in unmanipulated (control) broods, with no such effect in enlarged broods (Fig. 1). Specifically, in the control broods body mass increased at lower temperatures and reached ‘peak’ at the temperature of ca. 13.0 °C, and then decreased at higher temperatures. In the enlarged broods body mass steadily increased with raising temperature.

**Table 1:** The results of linear mixed models analysing the effects of a set explanatory variables on body mass and tarsus length of 14-day-old offspring

Model	Estimate (SE or CIs)	d.f	F	P
*Offspring body mass (N* = *2690)*				
Intercept	10.84 (0.06)			
**Treatment**	**−** **0.25 (0.06)**	**1, 451.2**	**19.98**	** < 0.001**
**Offspring sex**	**0.22 (0.03)**	**1, 2454.4**	**72.99**	** < 0.001**
Temperature	2.01 (0.70)	1, 109.6	1.04	0.31
Temperature^2^	− 2.01 (0.70)	1, 99.2	0.95	0.33
**Hatching date**	**0.09 (0.04)**	**1, 31.6**	**4.64**	**0.039**
**Body mass on day 2**	**0.26 (0.02)**	**1, 2513.4**	**281.20**	** < 0.001**
**Tarsus length**	**0.42 (0.02)**	**1, 2634.8**	**641.03**	** < 0.001**
**Treatment × temperature**	**−** **2.82 (0.83)**	**1, 369.0**	**11.72**	** < 0.001**
**Treatment × temperature**^**2**^	**2.89 (0.82)**	**1, 363.9**	**12.38**	** < 0.001**
Female identity	0.31 (0.25, 0.36)			
Foster female identity	0.51 (0.45, 0.57)			
Year	0.11 (0.00, 0.20)			
R^2^_marginal/conditional_	0.36/0.71			
*Offspring tarsus length (N* = *2694)*				
Intercept	16.10 (0.07)			
**Treatment**	**−** **0.09 (0.04)**	**1, 478.6**	**4.10**	**0.043**
**Offspring sex**	**0.41 (0.02)**	**1, 2400.0**	**519.16**	** < 0.001**
Temperature	0.86 (0.45)	1, 261.2	3.60	0.059
Temperature^2^	− 0.83 (0.46)	1, 219.3	3.30	0.071
**Body mass on day 2**	**0.18 (0.01)**	**1, 2587.9**	**245.42**	** < 0.001**
Female identity	0.26 (0.22, 0.31)			
Foster female identity	0.33 (0.28, 0.37)			
Year	0.18 (0.09, 0.28)			
R^2^_marginal/conditional_	0.17/0.61			

Full models (see Additional file 1: Table S2) included treatment (the level of this factor refers to enlarged nests) and offspring sex (the level of this factor refers to males) as categorical fixed factors, temperature and precipitation (both as linear and quadratic terms), hatching date, brood size, body mass on day 2 and tarsus length (only in body mass analysis) as covariates (all centred and standardised). Interactions between treatment and climatic variables were also tested. In all models, female identity, foster female identity and the year of study were random factors. Presented are reduced (final) models, with determined marginal and conditional R^2^, after the backward elimination of non-significant (if P > 0.1) interactions and covariates. Estimates of fixed and random factors are accompanied with SE and CIs, respectively. Significant terms (P < 0.05) are in bold

**Fig. 2:**
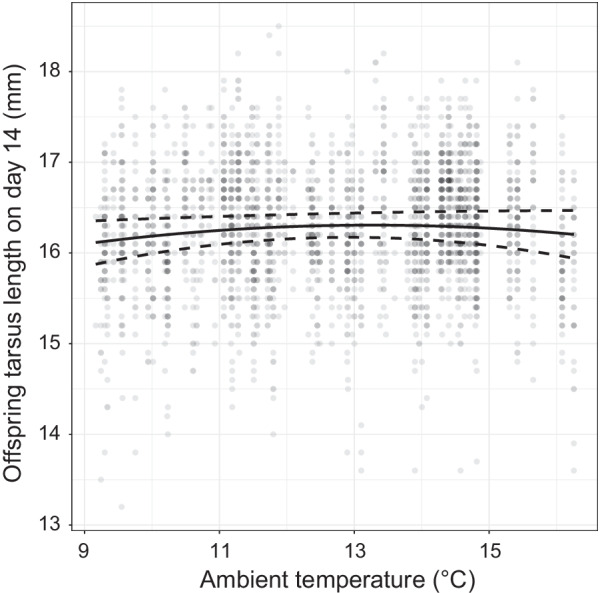
The effect of ambient temperature (quadratic term) on offspring tarsus length 14 days after hatching. The fitted regression line accompanied with 95% CI is presented, based on predicted values from the final model (Table 1)

Variation in tarsus length of 14-day-old nestlings was explained by brood size treatment, nestling sex and nestling body mass on day two (Table 1). However, we also found marginally non-significant effect of ambient temperature (quadratic term) (Table 1). The pattern of the relationship between offspring tarsus length and temperature was similar to the pattern observed for offspring body mass in control broods (Fig. 2), i.e., tarsus length increased at lower temperatures, reached ‘peak’ at ca. 13.0 °C, and then decreased at higher temperatures. Also, tarsus length of female nestlings was shorter compared to male nestlings (mean ± SE; 16.02 ± 0.02 mm vs. 16.48 ± 0.02 mm, respectively; Table 1). We found no evidence for an effect of precipitation on nestling mass or tarsus length on day 14 (see Additional file 1: Table S2).

## Discussion

Several studies on birds and other endotherms clearly demonstrated the impact of weather conditions during early postembryonic development on growth and long-term survival [35–37]. Here, we extend this research to show that ambient temperature during nestling stage can affect offspring phenotypes and get involved in potentially complex interactions with other environmental factors such as postnatal rearing conditions. Specifically, we showed that ambient temperature and brood size treatment interact to influence body mass of nestlings 14 days after hatching. This finding can have further fitness implications, since fledgling body mass is known to affect survival in blue tits [38] and other avian species [39–41].

A number of both observational and experimental studies showed that the nestlings grew faster and had larger body mass at fledgling in higher ambient temperatures [42, 43]. In contrast, some studies reported nestling growth rate and/or survival to decrease with increasing ambient temperature [36, 44, 45] in blue tits [24]. The inconsistencies between the studies may be explained by the fact that the sensitivity to weather conditions may differ among environments as well as life-history parameters. Indeed, in cold climate, higher ambient temperature might be beneficial for development [46], whereas higher ambient temperature in an already warm environment could be harmful to nestling development [13]. Our data suggests that environmental conditions (such as those generated via brood size manipulation) may affect the sensitivity to ambient temperature differentially. Specifically, in our study we observed that body mass steadily increased as temperature rose but only among nestlings from experimentally enlarged broods, where growth conditions were relatively unfavourable. In contrast, the body mass at the day 14 of nestlings raised in control nests showed a concave quadratic response to ambient temperature, indicating that the growth of developing nestlings is optimal across a range of 12–14 °C. Similar trend was observed in tarsus length—but it did not depend on the rearing conditions. In line with our predictions, we observed lower body mass among nestling from experimentally enlarged broods in most of the range of ambient temperatures in comparison to nestlings from unmanipulated, control broods. However, it is currently unclear why nestling raised in experimentally enlarged broods had higher body mass at higher temperatures (i.e. above 15.5 °C), it is possible that nestling from experimentally enlarged broods reach their optimum at higher temperatures. We can only speculate that these patterns can be attributed to the enhanced sibling competition for food in the enlarged broods where nestlings preferentially allocate resources towards the body mass increase. In line with previous studies we showed that optimal nestling growth and development at the post-hatching stage requires a certain range of ambient temperatures, outside of which physiological disturbances to growth and maturation are likely to arise [13, 21, 23, 36]. Moreover, our study highlights the importance of incorporating quadratic temperature effects on nestling development and physiology, with adverse effects occurring at the warm and cold end of the spectra, such measurements will provide crucial mechanistic depth to observed fitness consequences [13].

In the present study, we investigated the influence of temperature and rainfall on the body mass and structural size (tarsus length) over the development period, i.e. from hatching to fledging. A number of studies reported detrimental rainfall effects on nestling development [47–51]. Rainfall, also influenced growth patterns in the blue tits [52], probably because rainfall may reduce foraging efficiency of adults by limiting prey availability (mainly caterpillars [53]). For example, the feeding rate to offspring decreased in rainy days in great tits [47]. Therefore, it is difficult to explain the missing effect of rainfall on nestling body mass or tarsus length on day 14 in the present study. However, recent study on the blue tits [24] found that moderate rainfall had a positive, rather than a negative effect on nestling growth rate. Probably, relatively light rain may favour caterpillar movements and therefore increase food availability for tits [52], while intense rain may have the opposite effect.

## Conclusion

To sum up, these results indicate that the overall ambient temperature may have important consequences on offspring phenotypes. Importantly, our study suggests that the effects of ambient temperature on offspring body mass are likely to vary under different environmental settings (i.e., brood size manipulation treatment). Finally, our long-term study has another important implication: it suggests that the results of brood size manipulation can be biased by external conditions that remain uncontrolled by the experimenter. Thus we may expect the influence of changing weather conditions on experiments performed in the wild, including brood size manipulations.

## Methods

### Study species and site

The blue tit is a small, passerine bird from Paridae family. In our population females lay a single clutch per season, consisting on average of 11 eggs, and incubate the clutch alone for approximately two weeks. Nestlings are fed by both parents and nestlings fledge after 16–22 days [54]. After leaving the nest, young birds are fed by the parents for approximately another two weeks before reaching nutritional independence. The dataset considered in this paper includes years 2002–2012 and comprised 341 broods (2th day) and 334 broods (14th day) of 327 and 321 blue tit females, respectively (see details Additional file 1: Table S1). The study was carried out as a part of a long-term study on the reproductive biology of the blue tit population in southern part of Gotland island (57° 03′ N, 18° 17′ E). Wooden nest boxes were placed on a tree at about 1.3 m above ground level, and distributed approximately in a 30 × 50 m grid.

### Field techniques

The brood size manipulation experiment was performed across years 2002–2012. The number of breeding pairs ranged from 89 to 261 across the years (Additional file 1: Table S1). From the end of April we regularly inspected nest boxes to determine laying date, clutch size and hatching success. During each season (May–June), all breeding attempts were regularly monitored. Regular nest box checks (every 4 days) from the beginning of May established the date of clutch initiation. After hatching (hatching day = day 0 of nestling life), nests were visited on day 2 and 14 to obtain nestling measurements. To perform brood size manipulation, we created pairs of broods matched according to hatching date (± 1 day) and brood size (± 1 chick). Then, one randomly selected brood in each pair was enlarged (experimental nest) by adding three nestlings from a donor nests (not included in the analyses). This constitutes ca. 30% increase in brood size. The other brood within a pair was left with original number of nestlings (control nest). In addition before the brood size manipulation treatment, half of randomly chosen nestlings from each brood was exchanged between experimental and control broods (except for the season 2002). Such manipulation was performed for the purpose of other studies and as a result some chicks were raised by biological mothers and some by foster females (accounted for as female identity and foster female identity respectively in our analyses). All manipulations were performed on the second day after hatching. On that day, all nestlings were individually marked by clipping their nails and weighted with an electronic balance (to the nearest 0.1 g). Nestlings were weighted again on day 14 (hatching date = day 0). Tarsus length was measured on day 14 with an electronic calliper to the nearest 0.1 mm and each nestling was marked with an aluminium band. Adults were caught inside nest boxes or by mist–nets while feeding of 14-day-old nestlings (occasionally mist-netting was performed earlier, but never prior to day eight after hatching), weighed, measured and marked with an aluminium band. Blood samples (ca. 20 μl) were collected from all nestlings and adults and stored in 96% ethanol for further genetic analyses. DNA was extracted from blood samples with Chelex according to a standard protocol. Nestling sex was determined using P2 and P8 primers [55].

### Climatic factors

Daily temperature and precipitation records were obtained from the meteorological station at Hoburgen (56.92°N, 18.15°E; approximately 10 km from the main study areas). The data were accessed via the website of the Swedish Meteorological and Hydrological Institute (http://opendata-download-metobs.smhi.se/explore/?parameter=3). We calculated average daily temperature and daily sum of precipitation during a period from hatching day to day 14 after hatching. Importantly, both weather parameters were calculated for each nest separately. Both average ambient temperature and rainfall are extremely variable between years, but a clear variation is also observed within seasons (see Additional file 1: Figs. S1, S2).

### Statistical analysis

We fitted linear mixed models (LMMs) to analyse variation in offspring body mass (on day two and 14 after hatching) and tarsus length (on day 14) using the *lme4* package implemented in R environment [56]. All full models included brood size manipulation and offspring sex as categorical fixed factors, ambient temperature and daily sum of precipitation (both as linear and quadratic terms), hatching date, original brood size, body mass on day two (only in analyses of body mass and tarsus length on day 14) and tarsus length on day 14 (only in body mass analysis on day 14) as covariates. All covariates were centred and standardised, with the mean = 0 and unit = 1 SD. We also tested interactions between the brood size treatment and weather variables (temperature and precipitation). In all models female identity, foster female identity and the year of study were introduced as random factors. We reduced models using the backward elimination of non-significant (if P > 0.1) interactions and covariates (see Additional file 1 for full model details; Additional file 1: Table S2). We performed F-tests to test fixed effects using the *lmerTest* package [57], with the degrees of freedom approximated by the Satterthwaite method. We also calculated the marginal and conditional R^2^ for final (reduced) LMMs using the *MuMIn* package [58]. All models were checked for normality and heteroscedasticity of residuals. Only body mass on day two was log-transformed to meet those assumptions. We also tested for multicollinearity among explanatory variables (treated as linear predictors) using Variance Inflation Factor (VIF). Maximal VIF for all variables was smaller than two, indicating on a lack of serious problem with collinearity between independent variables. All tests were two-tailed and the significance level was set at P < 0.05. Sample sizes differed among analyses because of missing measurements, nest abandoned or predation. The raw means ± SE are presented throughout the results section.

## Supplementary Information

**Additional file 1**. **Table S1.** Annual nest-box occupancy by blue tits and the number of control and enlarged nests used in the experiment. **Table S2.** The results of initial (full) linear mixed models analysing the effects of a set explanatory variables on body mass and tarsus length of 14-day-old offspring. **Figure S1.** Average ambient temperature [oC] for the breeding season (May to June) across all years of the study 2002-2012, means ± SD. **Figure S2.** Average daily sums of precipitation [mm] for the breeding season (May to June) across all years of the study 2002-2012, means ± SD

## Data Availability

The datasets generated during and/or analysed during the current study will be available in the Dryad repository.
