# Supplementary material for: The interactive effect of ambient temperature and brood size manipulation on nestling body mass in blue tits: an exploratory analysis of a long-term study
Source: Front Zool. 2022 Feb 28;19:9. doi: 10.1186/s12983-022-00456-x (PMC8883686; doi:10.1186/s12983-022-00456-x)
Supplement: Supplementary file 1 — Additional file 1. Table S1. Annual nest-box occupancy by blue tits and the number of control and enlarged nests used in the experiment. Table S2. The results of initial (full) linear mixed models analysing the effects of a set explanatory variables on body mass and tarsus length of 14-day-old offspring. Figure S1. Average ambient temperature [oC] for the breeding season (May to June) across all years of the study 2002-2012, means ± SD. Figure S2. Average daily sums of precipitation [mm] for the breeding season (May to June) across all years of the study 2002-2012, means ± SD [file 12983_2022_456_MOESM1_ESM.docx]

**SUPPLEMENTARY MATERIAL**

Table S1 Annual nest-box occupancy by blue tits and the number of control and enlarged nests used in the experiment.

| **Year of study** | **Number of pair occupied nest-boxes (total number of nest-boxes each year is 2840)** | **Number of nests used in the experiment (control/enlarged)** |
| --- | --- | --- |
|  |  | 15/15 |
| 2003 | 149 | 15/32 |
| 2004 | 137 | 20/24 |
| 2005 | 138 | 18/18 |
| 2006 | 113 | 14/18 |
| 2007 | 92 | 14/16 |
| 2008 | 147 | 15/27 |
| 2009 | 136 | 10/17 |
| 2010 | 98 | 1/6 |
| 2011 | 179 | 13/16 |
| 2012 | 261 | 9/8 |

Table S2. The results of initial (full) linear mixed models analysing the effects of a set explanatory variables on body mass and tarsus length of 14-day-old offspring.

| **Model** | **Estimate (SE or CIs)** | **d.f.** | **F** | **P** |
| --- | --- | --- | --- | --- |
| *Offspring body mass (N = 2690)* |  |  |  |  |
| Intercept | 10.86 (0.07) |  |  |  |
| **Treatment** | **-0.25 (0.06)** | **1, 447.1** | **20.33** | **<0.001** |
| **Offspring sex** | **0.22 (0.03)** | **1, 2449.8** | **72.92** | **<0.001** |
| Temperature | 2.04 (0.74) | 1, 110.2 | 1.08 | 0.30 |
| Temperature^2^ | -2.07 (0.73) | 1, 97.2 | 1.02 | 0.32 |
| Precipitation | 0.04 (0.21) | 1, 22.3 | 0.01 | 0.93 |
| Precipitation^2^ | 0.02 (0.21) | 1, 26.0 | 0.00 | 0.97 |
| **Hatching date** | **0.10 (0.04)** | **1, 37.4** | **5.04** | **0.031** |
| Brood size | 0.02 (0.03) | 1, 472.3 | 0.49 | 0.48 |
| **Body mass on day 2** | **0.26 (0.02)** | **1, 2559.0** | **280.83** | **<0.001** |
| **Tarsus length** | **0.42 (0.02)** | **1, 2659.5** | **637.82** | **<0.001** |
| **Treatment × temperature** | **-2.78 (0.84)** | **1, 347.5** | **11.00** | **0.001** |
| **Treatment × temperature^2^** | **2.87 (0.84)** | **1, 339.7** | **11.75** | **<0.001** |
| Treatment × Precipitation | -0.05 (0.20) | 1, 390.2 | 0.06 | 0.81 |
| Treatment × Precipitation^2^ | -0.03 (0.21) | 1, 388.1 | 0.02 | 0.89 |
| Female identity | 0.31 (0.25, 0.36) |  |  |  |
| Foster female identity | 0.51 (0.45, 0.57) |  |  |  |
| Year | 0.15 (0.00, 0.22) |  |  |  |
| R^2^ _marginal/conditional_ | 0.36/0.71 |  |  |  |
| *Offspring tarsus length (N = 2694)* |  |  |  |  |
| Intercept | 16.12 (0.07) |  |  |  |
| **Treatment** | **-0.09 (0.04)** | **1, 464.1** | **4.85** | **0.028** |
| **Offspring sex** | **0.41 (0.02)** | **1, 2398.0** | **519.72** | **<0.001** |
| Temperature | 0.94 (0.57) | 1, 222.8 | 2.49 | 0.12 |
| Temperature^2^ | -0.97 (0.58) | 1, 188.0 | 2.46 | 0.12 |
| Precipitation | -0.0001 (0.17) | 1, 46.7 | 0.02 | 0.89 |
| Precipitation^2^ | 0.01 (0.17) | 1, 48.2 | 0.11 | 0.74 |
| Hatching date | 0.06 (0.04) | 1, 64.3 | 2.44 | 0.12 |
| Brood size | -0.01 (0.02) | 1, 504.8 | 0.30 | 0.58 |
| **Body mass on day 2** | **0.18 (0.01)** | **1, 2584.0** | **246.18** | **<0.001** |
| Treatment × temperature | -0.34 (0.65) | 1, 375.4 | 0.28 | 0.60 |
| Treatment × temperature^2^ | 0.40 (0.65) | 1, 368.6 | 0.38 | 0.54 |
| Treatment × Precipitation | 0.04 (0.16) | 1, 410.2 | 0.06 | 0.80 |
| Treatment × Precipitation^2^ | -0.12 (0.16) | 1, 433.8 | 0.51 | 0.47 |
| Female identity | 0.26 (0.22, 0.30) |  |  |  |
| Foster female identity | 0.32 (0.28, 0.37) |  |  |  |
| Year | 0.19 (0.09, 0.28) |  |  |  |
| R^2^ _marginal/conditional_ | 0.17/0.62 |  |  |  |

Full models included treatment (the level of this factor refers to enlarged nests) and offspring sex (the level of this factor refers to males) as categorical fixed factors, temperature and precipitation (both as linear and quadratic terms), hatching date, brood size, body mass on day 2 and tarsus length (only in body mass analysis) as covariates (all centred and standardised). Interactions between treatment and climatic variables were also tested. In all models female identity, foster female identity and the year of study were random factors. Presented are initial models, with determined marginal and conditional R^2^. Estimates of fixed and random factors are accompanied with SE and CIs, respectively. Significant terms (P < 0.05) are in bold.


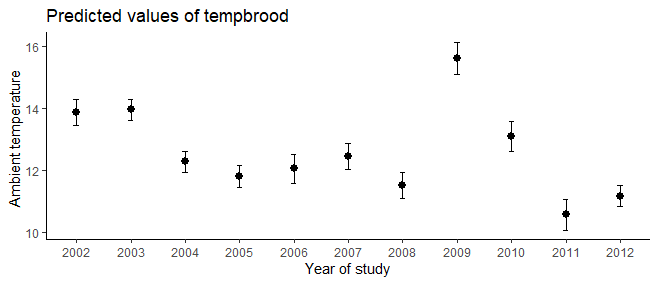


Figure S1. Average ambient temperature [oC] for the breeding season (May to June) across all years of the study 2002-2012, means±SD.


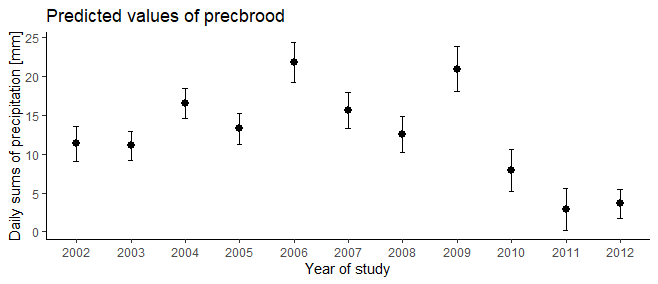


Figure S2. Average daily sums of precipitation [mm] for the breeding season (May to June) across all years of the study 2002-2012, means±SD.
